# Is Cardiorespiratory Fitness Related to Cardiometabolic Health and All-Cause Mortality Risk in Patients with Coronary Heart Disease? A CARE CR Study

**DOI:** 10.1186/s40798-018-0138-z

**Published:** 2018-05-30

**Authors:** Simon Nichols, Claire Taylor, Richard Page, Anna Kallvikbacka-Bennett, Fiona Nation, Toni Goodman, Andrew L. Clark, Sean Carroll, Lee Ingle

**Affiliations:** 1 Centre for Sport and Exercise Science, Sheffield Hallam University, Collegiate Hall, Collegiate Crescent, Sheffield, S10 2BP UK; 2 Carnegie School of Sport, Leeds Beckett University, Fairfax Hall, Headingley Campus, Leeds, LS6 3QS UK; 3 Sport Health and Exercise Science, Don Building, University of Hull, Cottingham Road, Hull, HU6 7RX UK; 4 Academic Cardiology, Castle Hill Hospital, Castle Road, Cottingham, HU16 5JQ UK; 5 City Health Care Partnership CIC, East Riding Community Hospital, Swinemoore Lane, Beverley, HU17 0FA UK

**Keywords:** Coronary Heart Disease, Cardiac Rehabilitation, Cardiometabolic Health, Exercise Training, Atherosclerosis, VO_2peak_, Maximal Cardiopulmonary Exercise Testing, CALIBER 5-year risk

## Abstract

**Background:**

Higher cardiorespiratory fitness (CRF) is associated with lower morbidity and mortality in patients with coronary heart disease (CHD). The mechanisms for this are not fully understood. A more favourable cardiometabolic risk factor profile may be responsible; however, few studies have comprehensively evaluated cardiometabolic risk factors in relation to CRF amongst patients with CHD. We aimed to explore differences in cardiometabolic risk and 5-year all-cause mortality risk in patients with CHD who have low, moderate, and high levels of CRF.

**Methods:**

Patients with CHD underwent maximal cardiopulmonary exercise testing, echocardiogram, carotid intima-media thickness measurement, spirometry, and dual X-ray absorptiometry assessment. Full blood count, biochemical lipid profiles, high-sensitivity (hs) C-reactive protein, and NT-proBNP were analysed. Patients were defined as having low, moderate, or high CRF based on established prognostic thresholds.

**Results:**

Seventy patients with CHD (age 63.1 ± 10.0 years, 86% male) were recruited. Patients with low CRF had a lower ventilatory anaerobic threshold, peak oxygen pulse, post-exercise heart rate recovery, and poor ventilatory efficiency. The low CRF group also had higher NT pro-BNP, hs-CRP, non-fasting glucose concentrations, and lower haemoglobin and haematocrit. Five-year mortality risk (CALIBER risk score) was also greatest in the lowest CRF group (14.9%).

**Conclusions:**

Practitioners should interpret low CRF as an important clinical risk factor associated with adverse cardiometabolic health and poor prognosis, study registry; www.researchregistry.com.

## Key Points

1. Low cardiorespiratory fitness is associated with the poorest cardiometabolic health in patients with coronary heart disease.
2. Five-year risk of all-cause death and NT-proBNP are highest amongst coronary heart disease patients with the lowest cardiorespiratory fitness, even when left ventricular ejection fraction is preserved.
  Longer-term or higher intensity exercise-based cardiac rehabilitation programmes that closely monitor cardiovascular risk factors may be warranted for coronary heart disease patients who have low cardiorespiratory fitness.

## Background

Cardiorespiratory fitness (CRF) or VO_2peak_ predicts all-cause and cardiovascular (CV) mortality in patients with coronary heart disease (CHD) [1–3]. We have shown that in patients with CHD, low CRF confers the highest mortality risk (54%) over a 14-year period when compared to those categorised as having moderate (31%) or high CRF [17%] [4]. Improvements in CRF resulting from exercise-based cardiac rehabilitation (CR) may reduce mortality in patients who have low CRF [4–6].

Whilst higher CRF is a strong predictor of better survival outcomes, the mechanisms responsible for this association are not fully understood. Few studies have comprehensively profiled the cardiometabolic health status of patients with CHD in relation to established CRF categories. Higher all-cause mortality in patients with CHD may not be entirely attributable to older age, cardiovascular disease (CVD) severity, and more comorbidities [7]. Unidentified and potentially treatable cardiometabolic risk factors may cause the divergence in mortality rates observed between patients with high and low CRF.

Biomarkers of cardiac dysfunction, inflammation, cardiac autonomic function, arterial plaque status, renal function, blood oxygen carrying capacity, and metabolic control have received little attention in relation to CRF in patients with CHD. Where limited data does exist, CRF is often reported as estimated metabolic equivalents (METS) from treadmill or cycle ergometer workloads [2, 4, 6]. Fitness estimation may be inaccurate in patients with CHD, could result in individual patients being inappropriately assigned to a specific CRF category and, attenuate any prognostic signal [8]. The association between cardiometabolic health and CRF should be investigated using ‘gold-standard’ maximal cardiopulmonary exercise testing (CPET).

This cross-sectional investigation aimed to assess differences in CV and cardiometabolic health amongst patients with CHD when characterised as have low, moderate, or high levels of CRF. We also investigated differences in estimated 5-year risk of death using the validated CALIBER composite scoring system [9].

## Methods

### Study Design

Data for this study were taken from the Cardiovascular and cardiorespiratory Adaptations to Routine Exercise-based Cardiac Rehabilitation study (CARE CR). Ethical approval for the study was given by the Humber Bridge NHS Research Ethics Committee-Yorkshire and the Humber (12/YH/0278). The study is registered with www.researchregistry.com (researchregistry3548). All procedures were conducted in accordance with the ethical standards outlined in the 1964 Helsinki declaration and its later amendments.

The methodology for this study has been reported elsewhere [10]. In brief, patients were recruited following a recent hospital admission and referral to CR for stable angina, myocardial infarction (MI), coronary artery bypass graft (CABG) surgery, or elective percutaneous coronary intervention (PCI). After giving verbal consent, patients were invited for assessment at Academic Cardiology, Castle Hill Hospital, Kingston-Upon-Hull, United Kingdom, where written informed consent was obtained.

### Resting Haemodynamics, Anthropometry, and Body Composition

Resting heart rate (HR) was determined at the end of 15 min, semi-supine rest using a 12-lead ECG (GE Healthcare, Buckinghamshire, UK). Left arm brachial blood pressure was recorded using an ECG-gated automated blood pressure (BP) cuff (Tango, SunTech Medical, Eynsham, UK).

Stature (cm) was measured using a Leicester Height Measure (SECA, Birmingham, UK) with patients standing, without footwear, in the Frankfort plane with their heels and head positioned to the back of the stadiometer. Waist circumference measurements were taken 1 cm above the iliac crest [11], and hip measurements were taken from the widest aspect of the buttocks using an inflexible tape measure. Both measurements were recorded in centimetre, and a ratio of the two was calculated to determine waist to hip circumference ratio [11].

Body composition was analysed using dual X-ray absorptometry [DXA] (Lunar iDXA, GE Healthcare, Buckinghamshire, UK). Total body mass (kg), lean body mass (kg), total fat (%), android fat (%), and android gynoid ratio were determined using the Lunar iDXA’s integrated software. DXA-derived total body mass was used to determine body mass index (BMI; kg.m^− 2^).

### Echocardiogram

Standard 2D, M-mode echocardiogram techniques were used to determine left ventricular (LV) function. LV ejection fraction (LVEF) was calculated using the Simpson’s method from measurements of end-diastolic and end-systolic volumes on apical 4-chamber and 2-chamber 2D views following the guidelines of Schiller and colleagues [12]. LV systolic dysfunction was diagnosed if LVEF was ≤ 45%.

### Carotid Intima-Media Thickness and Carotid Plaque Measurement

Carotid intima-media thickness (C-IMT) and carotid plaque measurements were obtained using the Panasonic CardioHealth Station (Panasonic Biomedical Sales Europe BV, Leicestershire, UK) which has low measurement variability [13, 14]. Measurements were taken using previously outlined methods [10, 13] from a 1 cm segment of the common carotid artery (CCA) located 1 cm proximally from the carotid bifurcation. Measurements from the right and left CCA were taken in the longitudinal plane at anterior (right CCA 150°; left CCA 210°), lateral (right CCA 120°; 230°), and posterior (right CCA 90°; left CCA 270°) angles, relative to ground. Each C-IMT measurement was determined from the average of 24 digital distance markers automatically placed between the intimal and medial boundaries and reported to three decimal places. Carotid artery plaques were measured in the longitudinal plane. The largest plaque was taken as the representative measure of the patient’s carotid plaque status and reported as being either < 1.0 mm, ≥ 1.0 to < 3.0 mm or ≥ 3.00 mm.

### Blood Samples

Blood samples were taken from a vein at the antecubital fossa and collected in ethylenediaminetetraacetic acid (EDTA), potassium oxalate, and serum separating tubes (SST). Full blood count, (haematocrit, haemoglobin, and neutrophil and lymphocyte count) and estimated glomerular filtration rate (eGFR) were analysed on the day of collection using a registered National Health Service (NHS) pathology lab (Castle Hill Hospital, Hull, United Kingdom). A further EDTA and potassium oxalate tube were placed in a refrigerated (4 °C) centrifuge at 3000 rpm, for 15 min immediately after the blood draw. Samples collected in SST tubes were allowed to clot for 30 min prior to being centrifuged under the same conditions.

The ABX Pentra 400 biochemistry auto analyser (Horiba, Montpellier, France) was used to analyse serum triglycerides, total cholesterol, high-density lipoprotein cholesterol (HDL), plasma glucose, and high-sensitivity C-reactive protein (hs-CRP) in duplicate. Calibration and quality controls were conducted in accordance with manufacturer’s guidelines. Low-density lipoprotein (LDL) was estimated using the Friedewald equation [15].

### Cardiopulmonary Exercise Testing and Assessment of Physical Activity

After 3 min of seated rest, CPET was conducted following the modified Bruce treadmill protocol (GE Healthcare, Buckinghamshire, UK) [16] in accordance with established guidelines [17–20]. A 12-lead ECG was monitored continuously throughout the test. An ECG-gated automated BP measurement was recorded at the start of CPET and at the second minute of a test stage until the end of the test. RPE scores were recorded at peak exercise. HR was recorded at 1, 2, 3, and 6 min during the passive seated recovery period.

Breath-by-breath gas exchange data were collected using an Oxycon Pro metabolic cart (Jaeger, Hoechburg, Germany). VO_2peak_ was defined as the mean VO_2_ (ml) over the last 30 s of the CPET. VO_2peak_ was adjusted for both body mass and lean (DXA-derived) body mass (ml/kg/min). The ventilatory anaerobic threshold (VAT) was determined using the V-slope method [21] by two independent investigators. Where agreement on VAT scores was not met, the results were discussed with a third independent investigator and a consensus was reached. The VAT was determined using the average of the middle 5 of 7 consecutive breaths (excluding the highest and lowest measures) and reported standardised to patient body mass (ml/kg/min). The oxygen uptake efficiency slope (OUES), VE/VCO_2_ slope, and oxygen pulse (VO_2_/HR) were calculated as previously described [19]. Self-reported weekly physical activity levels were obtained by asking patients if they participated in either 150 min of moderate physical activity, 75 min of vigorous physical activity, or both.

### Determination of Cardiorespiratory Fitness Categories

Patients were categorised into three groups based on their VO_2peak_. We converted VO_2peak_ into metabolic equivalents (METs) by dividing by 1 MET (estimated to be equivalent to a VO_2_ of 3.5 ml/kg/min) to allow for easier comparisons with other studies. Low CRF was defined as a peak MET value of < 5 for men and < 4 for women; high CRF was defined as peak MET > 7 for men and > 6 for women. These thresholds were based on previously published prognostic thresholds [4–6, 22, 23].

### Prognosis—CALIBER 5-year Risk Score

A 5-year risk of all-cause mortality was calculated for each patient using the comprehensive online (www.caliberresearch.org/model) CALIBER 5-year risk score [9]. This model does not include any CRF measurements in its calculation. A full list of included variables can be found in Table 1. Five-year risk of all-cause mortality was reported as a percentage to one decimal place.

**Table 1:** Variables Included in the CALIBER 5-Year Risk Score

Categorical Variables	Continuous Variables
Sex	Age (years)
Belongs to most deprived quintile	Total cholesterol (mmol/L)
CAD diagnosis and severity	HDL (mmol/L)
Interventions (last 6 months)	Heart rate (beats per minute)
Smoking status	Creatinine (micromol/L)
Hypertension/BP lowering medication	White cell count (10^9/L)
Diabetes	Haemoglobin (g/dl)
Heart failure	
Peripheral arterial disease	
Atrial fibrillation	
Stroke	
Chronic renal disease	
COPD	
Cancer	
Chronic liver disease	
Depression	
Anxiety	

*CAD* coronary artery disease, *BP* blood pressure, *COPD* chronic obstructive pulmonary disease, *HDL* high-density lipoprotein

### Statistical Analysis

Statistical analysis was performed using SPSS version 22 (IBM, New York, USA). The Shapiro-Wilk test and histograms were used to assess normality. Categorical data are reported as percentages. Continuous normally distributed variables are displayed as mean with 95% confidence intervals (95% CI) or standard deviation (±) where specified. One-way analysis of variance (ANOVA), one-way analysis of covariance (ANCOVA), and chi-squared analysis were used to assess differences between CRF groups. The only covariate considered in analysis was age as sex was used in assigning patients to their CRF category.

Statistical significance was set at *p* < 0.05. Partial eta squared (η_p_^2^) effect sizes were used to report the magnitude of group differences. Effect sizes of 0.01, 0.06, and 0.14 denoted small, moderate, and large effect sizes, respectively [24]. Pearson correlations were used to assess the strength of the relationship between CRF category and CALIBER 5-year risk of death. An *r* value of < 0.25, 0.26 to 0.50, 0.51 to 0.75, and, > 0.75 were considered weak, moderate, fair, and strong associations, respectively [25].

## Results

### Cohort Characteristics

Seventy patients were recruited. Sixteen (23%) patients had sustained an ST-elevation myocardial infarction (STEMI), 22 (31%) had sustained a non-STEMI, and 19 (27%) had undergone elective PCI. Seven patients (10%) were medically managed for stable angina, and six (9%) had undergone CABG. The mean age of the cohort was 63.1 ± 10.0 years (BMI 29.2 kg^.^m^− 2^ ± 4.0; 86% male). The mean LVEF was 55.0 ± 6.9%. The median time from cardiac event to consent was 54 days (range 22 to 220). 94% of patients were seen within 90 days.

Twenty-eight (40%), 32 (46%), and 10 (14%) of patients were defined as having high, moderate, and low levels of CRF, respectively. CRF group characteristics are shown in Table 2. Patients with low CRF were older (*p* < 0.001) and had a lower lean body mass than patients with moderate (*p* = 0.029) and high CRF (*p* = 0.002). Compared to patients with high (*p* < 0.001) and moderate CRF (*p* = 0.002), patients with low CRF also had a lower android gynoid ratio, higher resting HR (high CRF, *p* = 0.004;  moderate CRF, *p* = 0.042), and a larger proportion of patients suffering from type II diabetes (*p* = 0.028). Medications (Table 3) were comparable across all three groups with the exception of diuretics which were more commonly prescribed to patients in the low CRF group (*p* = 0.001).

**Table 2:** Patient Characteristics Expressed as Mean (95% Confidence Intervals)

Variable	High CRF, *n* = 28	Mod CRF, *n* = 32	Low CRF, *n* = 10	Partial eta squared	*p* value
Sex (% male)	26 (93)	27 (84)	7 (70)		0.199
Age (years)	56.3 (53.1, 59.4)^*✝^	67.2 (64.3, 70.2)^✝^	69.3 (64.1, 74.5)^*^	0.326	< 0.001^**^
BMI (kg.m^−2^)	29.0 (27.5, 30.5)	28.9 (27.5, 30.3)	30.9 (28.3, 33.4)	0.030	0.363
Waist circumference (cm)	101.3 (97.3, 105.4)	102.6 (98.7, 106.4)	108.1 (101.4, 114.9)	0.043	0.235
Waist to hip ratio (cm)	0.96 (0.93, 0.98)	0.98 (0.95, 1.00)	0.96 (0.92, 1.01)	0.018	0.553
Android fat %	45.0 (42.0, 48.0)	48.0 (45.2, 50.8)	47.9 (42.9, 52.9)	0.034	0.316
Total fat %	35.9 (32.2, 39.6)	37.3 (33.8, 40.7)	41.1 (34.8, 47.3)	0.029	0.371
Lean body mass (Kg)	55.0 (51.9, 58.1)^*✝^	50.2 (47.3, 53.1)^✝^	45.3 (40.1, 50.5)^*^	0.145	0.005^**^
Android/gynoid ratio	1.31 (1.24, 1.38)^*^	1.26 (1.20, 1.32)^x^	1.06 (0.95, 1.17)^*x^	0.182	0.001^**^
LVEF (%)	56.6 (54.0, 59.2)	54.3 (51.8, 56.6)	52.8 (48.5, 57.1)	0.043	0.232
Resting HR (bpm)	56 (52, 69)^*^	59 (56, 63)^x^	67 (61, 74)^*x^	0.118	0.015^**^
Resting SBP (mmHg)	130 (122, 137)	124 (117, 131)	138 (125, 150)	0.056	0.147
Resting DBP (mmHg)	85 (80, 89)	82 (77, 86)	73 (65, 81)	0.083	0.055
FEV_1_/FVC ratio	0.78 (0.75, 0.81)	0.75 (0.72, 0.78)	0.75 (0.70, 0.80)	0.042	0.237
Presenting diagnosis					
MI (%)	17 (61)	15 (47)	6 (60)		0.642
PCI (%)	8 (29)	11 (34)	1 (10)		
Angina (%)	2 (7)	3 (9)	2 (20)		
CABG (%)	1 (4)	3 (9)	1 (10)		
Past medical history					
Previous MI (%)	6 (21)	6 (19)	2 (20)		0.186
Type II diabetes (%)	3 (12)	6 (19)	5 (50)		0.028^**^
Atrial fibrillation (%)	0 (0)	2 (6)	1 (10)		0.269
Smoker (%)	1 (4)	1 (3)	2 (20)		0.238
Ex-smoker (%)	16 (57)	16 (50)	7 (70)		

*Mod* moderate, *BMI* body mass index, *LVEF* left ventricular ejection fraction, *HR* heart rate, *SBP* systolic blood pressure, *DBP* diastolic blood pressure, *FEV1/FVC* ratio of forced expiratory volume in 1 s to forced vital capacity, *MI* myocardial infarction, *PCI* percutaneous coronary intervention, *CABG* coronary artery bypass graft

**Significant group effect

*Significant difference between high CRF and low CRF

^✝^Significant difference between high CRF and moderate CRF

^x^Significant difference between mod CRF and low CRF

**Table 3:** Number (%) of Medications Taken by Patients

Medication	High CRF, *n* = 28	Mod CRF, *n* = 32	Low CRF, *n* = 10	*p* value
Aspirin (%)	27 (96)	31 (97)	10 (100)	0.838
Ticagrelor (%)	15 (54)	17 (53)	3 (30)	0.393
Clopidogrel (%)	10 (36)	8 (25)	3 (30)	0.665
Anti-coagulants (%)	0 (0)	1 (3)	1 (10)	0.263
Beta-blockers (%)	24 (86)	29 (91)	7 (70)	0.266
ACE-inhibitors (%)	19 (68)	16 (50)	7 (70)	0.291
Angiotensin receptor blockers (%)	0 (0)	5 (16)	1 (10)	0.096
Statins (%)	25 (89)	32 (100)	10 (100)	0.095
Diuretics (%)	1 (4)	1 (3)	5 (50)	0.001**
Calcium channel blockers (%)	4 (14)	8 (25)	4 (40)	0.502
Nitrates (%)	2 (7)	3 (9)	3 (30)	0.233

*ACE* angiotensin converting enzyme

**Significant group effect

### Cardiorespiratory Fitness and Physical Activity

Cardiorespiratory fitness and physical activity-related variables are shown in Table 4. Differences in VO_2peak_ standardised to lean body mass, VAT, VO_2_/HR, and exercise test duration were observed across all groups. Compared to patients with high CRF, VE/VCO_2_ slope and OUES were both higher amongst patients in the moderate and low CRF groups. Mean LVEF was not different across the groups and was not significantly correlated with peak METs (*r* = 0.147; *p* = 0.224). One patient in both the low and moderate CRF groups had a LVEF < 40%. Compared to patients with high and moderate CRF, patients with low CRF had the most impaired 1-min HR recovery, an indicator of cardiac autonomic function. The proportion of patients who reported participating in either 150 min of moderate (*p* = 0.011) or 75 min of vigorous physical activity per week was higher in the high CRF group.

**Table 4:** Cardiorespiratory Fitness and Physical Activity Characteristics Expressed as Mean (95% Confidence Intervals)

Variable	High CRF, *n* = 28	Mod CRF, *n* = 32	Low CRF, *n* = 10	Partial eta squared
VO_2peak_ (ml/kg/min)	28.5 (27.3, 29.7)^+^	20.7 (19.5, 21.8)^+^	14.9 (12.8, 16.9)^+^	–
VO_2peak_ (L/min)	2478.2 (2333.0, 2623.5)^+^	1749.0 (1613.1, 1884.9)^+^	1273.8 (1030.7, 1516.8)^+^	–
VO_2peak_-lean (ml/kg/min)	45.2 (43.4, 47.0)^+^	34.8 (33.2, 36.5)^+^	26.8 (23.8, 29.8)^+^	0.670
VAT (ml/kg/min)	20.7 (19.3, 22.1)^+^	14.6 (13.3, 15.9)^+^	11.2 (8.9, 13.6)^+^	0.494
VE/VCO_2_ slope	30.1 (28.2, 32.1)*^✝^	37.4 (35.6, 39.2)^✝^	38.5 (35.2, 41.7)^*^	0.354
VO_2_/HR (ml/beat)	17.0 (15.8, 18.2)^+^	13.8 (12.7, 14.9)^+^	11.3 (9.4, 13.3)^+^	0.311
OUES	2718.3 (2555.3, 2881.3)*^✝^	1963.5 (1811.1, 2116.0)^✝^	1699.0 (1426.2, 1971.7)^*^	0.485
eBR (%)	30.3 (23.6, 36.9)	28.1 (22.0, 34.3)	37.0 (26.0, 48.1)	0.028
Peak HR (bpm)	147 (141, 153)^*^^✝^	128 (122, 134)^✝^	119 (108, 129)^*^	0.308
Peak RER	1.13 (1.09, 1.12)^*^	1.09 (1.05, 1.12)^x^	0.97 (0.91, 1.04)*^x^	0.181
Peak RPE	18 (17, 19)	18 (17, 19)	17 (15, 18)	0.072
1 min HR recovery (bpm)	− 36 (− 32, − 40)^*^	− 30 (− 26, − 34)^x^	− 18 (− 11, −25)*^x^	0.209
2 min HR recovery (bpm)	− 54 (− 50, − 59)^+^	− 45 (− 40, − 49)^+^	− 32 (− 25, −38)^+^	0.312
3 min HR recovery (bpm)	− 60 (− 56, − 65)^+^	− 49 (− 45, − 53)^+^	− 37 (− 30, −44)^+^	0.359
6 min HR recovery (bpm)	− 67 (− 62, − 71)^+^	− 54 (− 50, − 58)^+^	− 41 (− 33, − 48)^+^	0.377
Exercise test duration (s)	963.2 (916.3, 1010.1)^+^	747.8 (703.9, 791.6)^+^	488.3 (409.8, 566.8)^+^	0.635
METs	8.1 (7.8, 8.5)^+^	5.9 (5.6, 6.2)^+^	4.3 (3.7, 4.8)^+^	–
Maximal CPET (%)	26 (93)	26 (81)	6 (60)	0.058
Achieves 150 min of moderate activity per week (%)	18 (64)^+^	9 (28)	5 (50)	0.011^*^^*^
Achieves 75 min of vigorous activity per week (%)	7 (25)^+^	1 (3)	0 (0)	0.013^**^

*VO*_*2peak*_ peak oxygen uptake, *VAT* ventilatory anaerobic threshold, *VE/VCO*_*2*_ ventilatory efficiency with respect to CO_2_ elimination, *VO*_*2*_*/HR* oxygen pulse, *OUES* oxygen uptake efficiency slope, *eBR* estimated breathing reserve, *HR* heart rate, *bpm* beats per minute, *RER* respiratory exchange ratio, *RPE* rating of perceived exertion; *S* seconds, *METs* metabolic equivalents

**Significant group effect

*Significant difference between high CRF and low CRF

^✝^Significant difference between high CRF and moderate CRF

^x^Significant difference between mod CRF and low CRF

^+^Significantly different from all other groups

### Blood Biomarkers and Cardiovascular Risk

Results of blood biochemical analyses are displayed in Table 5. NT-proBNP was inversely associated with CRF (*r* = -0.414) and was highest amongst patients with low CRF. Four (40%) patients with low CRF had an NT-proBNP > 400 pg/ml compared to eight (25%) and one (4%) in the moderate and high CRF groups. One patient in the low CRF group without previously diagnosed chronic heart failure (CHF) had an NT-proBNP result > 2000 pg/ml with a mildly reduced LVEF (45%), sinus rhythm, and with an eGFR of 73 mL/min/1.73 m^2^. Lipid profiles did not differ between groups. Non-fasting plasma glucose concentrations in the low CRF group were higher than the moderate CRF group (*p* = 0.008). hs-CRP was also highest in the low CRF group. Haematocrit and haemoglobin differed between all groups.

**Table 5:** Blood Biomarkers Expressed as Mean (95% Confidence Intervals)

Variable	High CRF, *n* = 28	Mod CRF, *n* = 32	Low CRF, *n* = 10	Partial eta squared	*p* value
NT-proBNP (ng/L)	86.1 (11.4, 1428.0) *^✝^	217.0 (33.9, 1916.0)^✝^	464.0 (26.9, 2735.0)*	-	< 0.001**
Total Chol (mmol/L)	3.6 (3.2, 3.9)	3.7 (3.4, 4.0)	3.9 (3.3, 4.5)	0.017	0.579
LDL Chol (mmol/L)	1.7 (1.4, 1.9)	1.8 (1.6, 2.1)	1.9 (1.4, 2.3)	0.019	0.529
HDL Chol (mmol/L)	1.2 (1.0, 1.3)	1.2 (1.0, 1.3)	1.4 (1.2, 1.6)	0.052	0.176
TC/HDL ratio	3.2 (2.9, 3.5)	3.3 (3.0, 3.6)	3.0 (2.5, 3.6)	0.01	0.713
Triglycerides (mmol/L)	1.6 (1.3, 1.9)	1.5 (1.2, 1.8)	1.4 (0.9, 2.0	0.011	0.691
Glucose (mmol/L)	5.7 (3.3, 13.8)	5.2 (4.1, 22.2)^x^	6.4 (5.5, 16.9)^x^	-	0.012**
hs-CRP (mg/L)	1.4 (0.3, 2.5)*	2.8 (1.7, 3.8)	4.2 (2.3, 6.2)*	0.099	0.033**
Neutrophil/lymphocyte ratio	2.4 (2.1, 2.8)	2.8 (2.4, 3.1)	3.3 (2.7, 3.8)	0.084	0.052
Haematocrit (%)	0.423 (0.410, 0.435)^+^	0.405 (0.393, 0.417)^+^	0.376 (.355, 0.397)^+^	0.180	0.001**
Haemoglobin (g/L)	145.5 (140.7, 150.2)^+^	138.2 (133.7, 142.6)^+^	125.6 (117.6, 133.6)^+^	0.219	< 0.001**
eGFR (mL/min/1.73 m^2^)	80.2 (75.2, 85.3)	76.2 (71.4, 80.9)	71.0 (62.6, 79.4)	0.053	0.160

*NT-proBNP* N-terminal prohormone brain natriuretic peptides, *Chol* cholesterol, *LDL* low-density lipoproteins, *HDL* high-density lipoproteins, *hs-CRP* high-sensitivity C-reactive protein, *eGF* estimated glomerular filtration rate

**Significant group effect

*Significant difference between high CRF and low CRF

✝Significant difference between high CRF and moderate CRF

^x^Significant difference between moderate CRF and low CRF

^+^Significantly different from all other groups

### Carotid Intima-Media Thickness

**Fig. 1:**
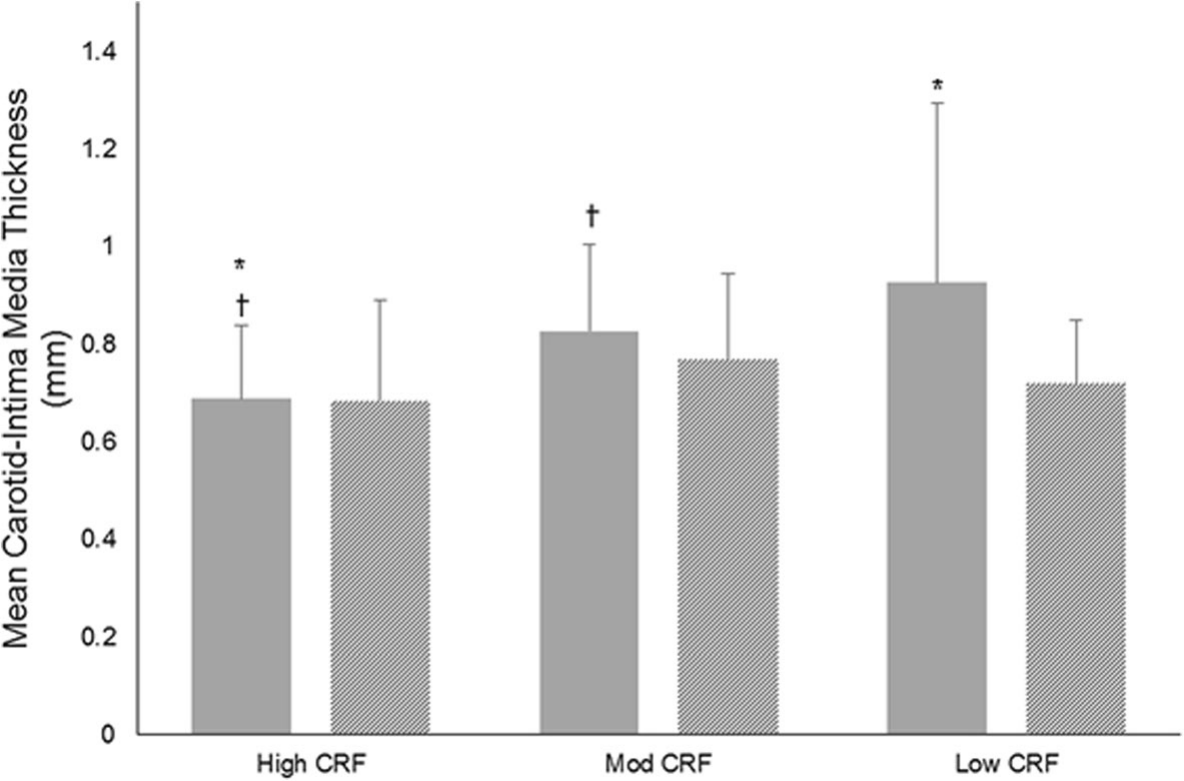
Carotid intima-media thickness measurements in low, moderate, and high cardiorespiratory fitness groups. Mean left-sided carotid intima-media thickness measurements (solid grey bars) were higher in the low and moderate cardiorespiratory fitness categories. Mean right-sided carotid intima-media thickness (lines) did not differ between groups. Mod = moderate; CRF = cardiorespiratory fitness *Significant difference between high CRF and low CRF; ✝Significant difference between high CRF and moderate CRF

**Fig. 2:**
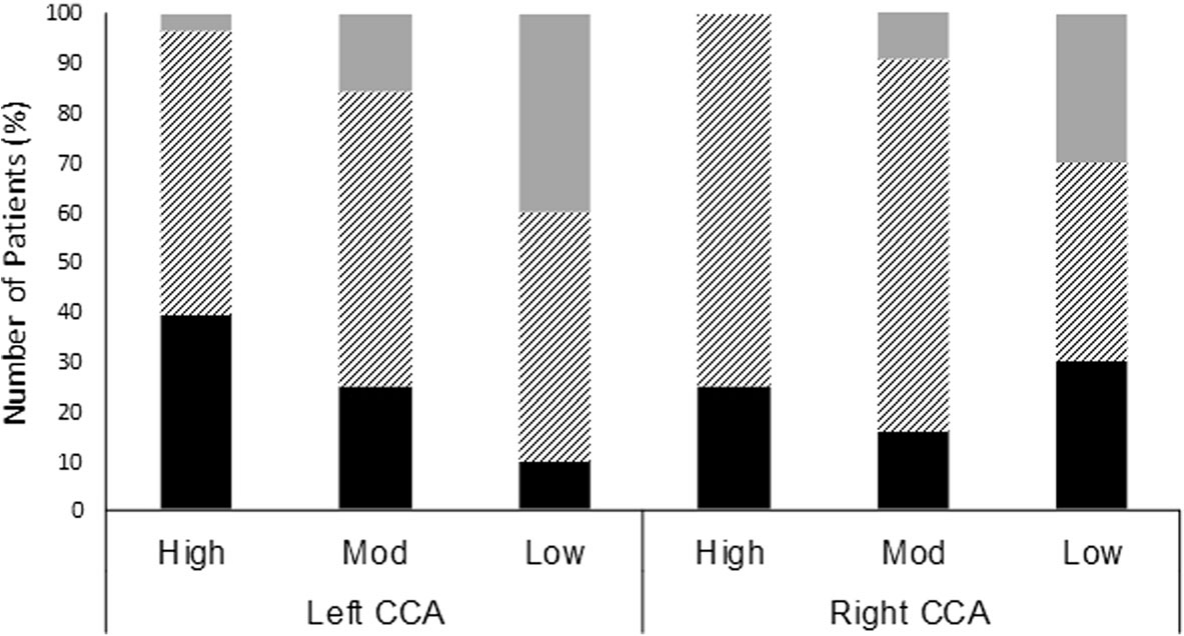
Left- and right-side common  carotid artery plaque severity in patients with high, moderate, and low cardiorespiratory fitness. Black bars indicate the proportion of patients with plaques < 1 mm and diagonal lines indicate the proportion of patients with plaques between 1 and 3 mm. Grey bars indicate the proportion of patients with plaques > 3 mm. Mod = moderate; CCA = common carotid artery

A moderate inverse correlation between left-sided C-IMT measurements and CRF was observed (*r* = − 0.382; *p* = 0.001) with larger measurements seen in the low (0.926; 95% CI 0.797, 1.054; *p* = 0.002) and moderate CRF groups (0.827; 95% CI 0.755, 0.898; *p* = 0.011) compared to the high CRF group [0.689; 95% CI 0.612, 0.765] (Fig. 1). There was no significant correlation between right sided C-IMT and CRF (*r* = − 0.210; *p* = 0.080). The proportion of patients with left-sided plaque score < 1 mm (Fig. 2) increased with each CRF group (low 10%; moderate 25%; high 39%). The proportion of patients exhibiting large left-sided plaque scores (> 3 mm) decreased across each group (low 40%; moderate 16%; high 4%; *p* = 0.047). Patients with moderate and low CRF had incrementally higher proportions of patients with right-sided plaque scores > 3 mm (moderate CRF 9.4%; low CRF 30%) compared to patients in the high CRF who had no plaques > 3 mm (Fig. 2; *p* = 0.032).

### All-Cause Mortality Estimation

**Fig. 3:**
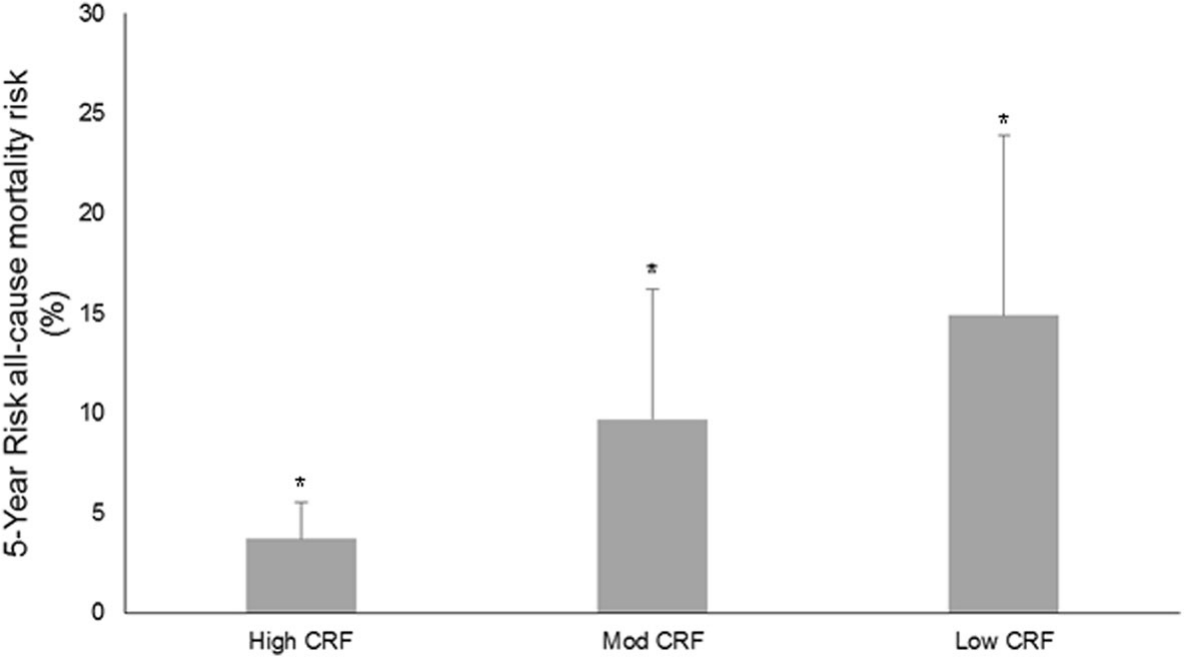
CALIBER 5-year all-cause mortality risk scores were incrementally higher across the three cardiorespiratory fitness groups. Mod = moderate; CRF = cardiorespiratory fitness +Significantly different from all other groups

Differences in CALIBER 5-year risk (Fig. 3) were observed between all groups. Estimated risk was highest in the low CRF group (14.9; 95% CI 11.4, 18.5%). The moderate group had a 9.7% 5-year risk of death (95% CI 7.7, 11.7%) whilst high CRF was associated with lowest 5-year risk of death (3.7%; 95% CI 1.6, 5.8%). There was a negative association between peak METS and CALIBER 5-year risk score (*r* = − 0.538; *r*^2^ = 0.289; *p* = < 0.001).

### Statistical Adjustment for Age

Differences in left-sided mean C-IMT (*p* = 0.274; η_p_^2^ = 0.039) became non-significant following age adjustment. Statistically significant differences for all other variables remained unaltered after statistical adjustment for age.

## Discussion

To our knowledge, this is the first study to comprehensively profile the cardiometabolic and CV health status of patients with CHD according to prognostically verified CRF categories [4–6, 22, 23]. Patients with the lowest CRF have the poorest integrated cardiorespiratory function and cardiometabolic health. Patients with low CRF may also have impaired autonomic function and worse CV disease severity. Low CRF was associated with the poorest CALIBER 5-year all-cause mortality risk and ‘high risk’ status (defined as 3% annual mortality risk [26]) despite the risk model not including CRF-based measurements. The CALIBER model includes an extensive set of all-cause mortality predictor variables, including sociodemographics, CHD severity and phenotype, primary CVD risk factors, CVD and non-CVD comorbidities, psychosocial factors, and biomarkers. This provides further evidence that CRF and other CPET-derived variables should be treated as ‘clinical vital sign’ [27].

An important finding of this study was that NT-proBNP levels and VE/VCO_2_ slopes were highest in the moderate and low CRF groups. This suggests a greater degree of cardiac dysfunction, ventilatory perfusion mismatch, or heightened peripheral chemoreceptor sensitivity as seen in patients with CHF [28, 29]. NT-proBNP levels and VE/VCO_2_ slopes were higher even in the absence of significant resting LV impairment. It is possible that patients with CHD and low CRF have undiagnosed CHF or are in the early stages of its development. Future research is required to determine whether lower CRF in patients with CHD confers a greater risk of developing CHF. Closer monitoring for signs and symptoms of CHF may be needed for patients who enter CR and have low CRF.

The prognostic value of a high VE/VCO_2_ slope and/or NT-proBNP has been well-described in CHF [30–32]. However, the value of VE/VCO_2_ slope and NT-proBNP levels has also been shown in CHD. Elevated values predict all-cause mortality [33], cardiovascular mortality (~ 5 year follow-up), and the development of CHF and stroke [34]. A high VE/VCO_2_ is independently associated with high NT-proBNP levels, as well as ventricular remodelling [35]. Our findings suggest that patients with both low and moderate CRF levels are at greatest risk of having such adverse health outcomes, corroborated by their higher CALIBER 5-year risk.

Traditional risk factors such as angina symptoms, prior MI, atrial fibrillation, claudication, type II diabetes, CHF, and smoking history do not entirely explain the increased mortality risk associated with having low CRF. Physical activity participation and higher CRF have been established as important independent causative factors for recurrent events [7]. In our study, patients defined as having high CRF reported participating in regular physical activity and had the lowest 5-year risk of death. This was accompanied by a lower resting HR and a quicker post-exercise HR recovery, indicators of autonomic function which also carry prognostic value. Interestingly however, no differences were evident in standard anthropometric indices (BMI or waist circumference) or in DXA-derived body fat content across the CRF groups.

The link between cardiometabolic profile, body composition, and cardiovascular risk has been widely reported and there is often an emphasis on reducing overweight and obesity states in CR programmes. In our study, patients with higher CRF had a larger proportion of lean tissue and more favourable abdominal fat distribution. Low CRF was only associated with latent changes in body composition. Changes were not evident when assessing patients with routinely applied anthropometric techniques, such as BMI and waist circumference. Whilst overweight patients with CHD have been reported to benefit from a better prognosis than their leaner counterparts, [36], the association between higher BMI and reduced all-cause mortality may depend on a patients’ CRF. Those characterised as having low CRF appear to benefit from improved survival whilst fitter individuals do not [37, 38]. Our findings (incorporating criterion fitness and ‘fatness’ measures) suggest that maintaining or improving CRF may help lower recurrent cardiovascular risk in patients with CHD. However, our sample is relatively small and only 10 patients were identified as having a low CRF (*n* = 10). This may increase the likelihood of statistical error (type I/type II error) when comparing CRF groups. However, many of the effect sizes (ηp^2^) associated with differences in cardiometabolic risk were large. Furthermore, the 95% CI indicate distinct values for many cardiometabolic variables across the CRF groups. However, further research using a larger patient cohort is required to confirm if our findings are representative of the wider CHD population.

As reported by others [4, 6], our data shows that  patients with high CRF have lower CALIBER 5-year risk scores compared to patients with low and moderate CRF. In previous studies [4, 6], patients characterised as having high CRF were shown to have superior survival over a 15-year period. Barons and colleagues [6] reported that within their low CRF group, a history of MI, PCI, and angina were associated with the highest risk of death. Our data do not confirm this. However, similar to our findings, Taylor et al. [4] reported that prior MI is not more prevalent amongst patients with low CRF. It is possible that size and location of MI are responsible for differences in cardiac dysfunction and mortality risk.

Patients with a high CRF also had smaller left-sided C-IMT measurements compared to patients with moderate or low CRF. This may be because patients with a high CRF also had a better cardiometabolic profile, including high levels of self-reported physical activity. Exercise training can improve cardiovascular risk factor profiles in patients with CHD [39] and attenuate C-IMT and carotid plaque progression in as little as 6 to 12 months [40, 41]. It is however important to acknowledge that older age attenuated the relationship between C-IMT and CRF.

## Conclusions

Identification of patients with low CRF and CHD appears to be an effective means of identifying those at highest cardiometabolic risk, and increased risk of 5-year all-cause mortality. Exercise testing is widely applied in cardiological and CR environments. Longer-term, or higher intensity exercise-based CR programmes may help improve the cardiometabolic health of patients with CHD and low CRF.

## Abbreviations

±: Standard deviation
95% CI: 95% confidence Intervals
ANCOVA: Analysis of covariance
ANOVA: Analysis of variance
BMI: Body mass index
BP: Blood pressure
CABG: Coronary artery bypass graft
CCA: Common carotid artery
CHD: Coronary heart disease
CHF: Chronic heart failure
C-IMT: Carotid intima-media thickness
CPET: Cardiopulmonary exercise test
CR: Cardiac rehabilitation
CRF: Cardiorespiratory fitness
CV: Cardiovascular
CVD: Cardiovascular disease
DXA: Dual X-ray absorptiometry
ECG: Electrocardiogram
EDTA: Ethylenediaminetetraacetic acid
e-GFR: Estimated glomerular filtration rate
Hb: Haemoglobin
Hct: Haematocrit
HDL: High-density lipoproteins
HR: Heart rate
hs-CRP: High-sensitivity C-reactive protein
LDL: Low-density lipoproteins
LV: Left ventricular
LVEF: Left ventricular ejection fraction
METs: Metabolic equivalents
MI: Myocardial infarction
NHS: National Health Service
NT-proBNP: Terminal pro B type natriuretic peptide
VO_2_/HR: Oxygen pulse
OUES: Oxygen uptake efficiency slope
PCI: Percutaneous coronary intervention
RPE: Rating of perceived exertion
SST: Serum separating tube
VAT: Ventilatory anaerobic threshold
VE/VCO_2_ slope: Ventilatory efficiency with respect to CO_2_ elimination
VO_2_: Oxygen uptake
VO_2peak_: Peak oxygen uptake
η_p_^2^: Partial eta squared

## References

[1] Franklin BA, Lavie CJ, Squires RW, Milani RV (2013). Exercise-based cardiac rehabilitation and improvements in cardiorespiratory fitness: implications regarding patient benefit. Mayo Clin Proc.

[2] McAuley PA, Artero EG, Sui X, Lee D-C, Church TS, Lavie CJ (2012). The obesity paradox, cardiorespiratory fitness, and coronary heart disease. Mayo Clin Proc.

[3] Keteyian SJ, Brawner CA, Savage PD, Ehrman JK, Schairer J, Divine G (2008). Peak aerobic capacity predicts prognosis in patients with coronary heart disease. Am Heart J.

[4] Taylor C, Tsakirides C, Moxon J, Moxon J, Dudfield M, Witte KK (2016). Submaximal fitness and mortality risk reduction in coronary heart disease: a retrospective cohort study of community-based exercise rehabilitation. BMJ Open.

[5] Martin BJ, Arena R, Haykowsky M, Hauer T, Austford LD, Knudtson M (2013). Cardiovascular fitness and mortality after contemporary cardiac rehabilitation. Mayo Clin Proc.

[6] Barons MJ, Turner S, Parsons N, Griffiths F, Bethell H, Weich S, et al. Fitness predicts long-term survival after a cardiovascular event: a prospective cohort study. BMJ Open. 2015;5:e007772. 10.1136/bmjopen-2015-007772 10.1136/bmjopen-2015-007772 PMC4620170 26493455

[7] Mandic S, Myers J, Oliveira RB, Abella J, Froelicher VF (2010). Characterizing differences in mortality at the low end of the fitness spectrum in individuals with cardiovascular disease. Eur J Cardiovasc Prev Rehabil.

[8] Nichols S, Gleadall-Sidall DO, Antony R, Clark AL, Cleland JGF, Carroll S, et al. Estimated peak functional capacity; an accurate method for assessing change in peak oxygen consumption after cardiac rehabilitation? Clin Physiol Funct Imaging. 2017; Ahead of print 10.1111/cpf.12468 28857391

[9] Rapsomaniki E, Shah A, Perel P, Denaxas S, George J, Nicholas O (2014). Prognostic models for stable coronary artery disease based on electronic health record cohort of 102 023 patients. Eur Heart J.

[10] Nichols S, Nation F, Goodman T, Clark AL, Carroll S, Ingle L. CARE CR-cardiovascular and cardiorespiratory adaptations to routine exercise-based cardiac rehabilitation: a study protocol for a community-based controlled study with criterion methods. BMJ Open.2018;8:e019216. 10.1136/bmjopen-2017-019216. 10.1136/bmjopen-2017-019216 PMC5829840 29374670

[11] ACSM (2017). ACSM’s guidelines for exercise testing and prescription. 10th ed.

[12] Schiller NB, Shah PM, Crawford M, DeMaria A, Devereux R, Feigenbaum H (1989). Recommendations for quantitation of the left ventricle by two-dimensional echocardiography. American Society of Echocardiography Committee on standards, subcommittee on quantitation of two-dimensional echocardiograms. J Am Soc Echocardiogr.

[13] Nichols S, Milner M, Meijer R, Carroll S (2014). Ingle L.

[14] Vanoli D, Wiklund U, Lindqvist P, Henein M, Näslund U (2013). Successful novice’s training in obtaining accurate assessment of carotid IMT using an automated ultrasound system. European Heart Journal – Cardiovascular Imaging.

[15] Friedewald WT, Levy RI, Fredrickson DS (1972). Estimation of the concentration of low-density lipoprotein cholesterol in plasma, without use of the preparative ultracentrifuge. Clin Chem.

[16] Bruce RA, Kusumi F, Hosmer D (1973). Maximal oxygen intake and nomographic assessment of functional aerobic impairment in cardiovascular disease. Am Heart J.

[17] American Thoracic Society/American College of Chest Physicians (2003). ATS/ACCP statement on cardiopulmonary exercise testing. Am J Respir Crit Care Med.

[18] Taylor C, Nichols S, Ingle L (2015). A clinician’s guide to cardiopulmonary exercise testing 1: an introduction. Br J Hosp Med.

[19] Nichols S, Taylor C, Ingle L (2015). A clinician's guide to cardiopulmonary exercise testing 2: test interpretation. Br J Hosp Med.

[20] Balady GJ, Arena R, Sietsema K, Myers J, Coke L, Fletcher GF (2010). Clinician’s guide to cardiopulmonary exercise testing in adults: a scientific statement from the American Heart Association. Circulation.

[21] Beaver WL, Wasserman K, Whipp BJ (1986). A new method for detecting anaerobic threshold by gas exchange. J Appl Physiol.

[22] Kavanagh T, Mertens DJ, Hamm LF, Beyene J, Kennedy J, Corey P (2003). Peak oxygen intake and cardiac mortality in women referred for cardiac rehabilitation. J Am Coll Cardiol.

[23] Myers J, Prakash M, Froelicher V, Do D, Partington S, Atwood JE (2002). Exercise capacity and mortality among men referred for exercise testing. N Engl J Med.

[24] Richardson JTE (2011). Eta squared and partial eta squared as measures of effect size in educational research. Educational Research Review.

[25] Berg KE, Latin RW (2008). Essentials of research methods in health, physical education, exercise science, and recreation: Lippincott Williams & Wilkins.

[26] Montalescot G, Sechtem U, Achenbach S, Andreotti F, Arden C, Budaj A (2013). 2013 ESC guidelines on the management of stable coronary artery disease. The task force on the management of stable coronary artery disease of the European Society of Cardiology. Eur Heart J.

[27] Ross R, Blair SN, Arena R, Church TS, Després J-P, Franklin BA, et al. Importance of assessing cardiorespiratory fitness in clinical practice: a case for fitness as a clinical vital sign: a scientific statement from the American Heart Association. Circulation. 2016:65. CIR. 0000000000000461 10.1161/CIR.0000000000000461 27881567

[28] Poole DC, Hirai DM, Copp SW, Musch TI (2012). Muscle oxygen transport and utilization in heart failure: implications for exercise (in) tolerance. Am J Phys Heart Circ Phys.

[29] Clark AL, Poole-Wilson PA, Coats AJS (1996). Exercise limitation in chronic heart failure: central role of the periphery. J Am Coll Cardiol.

[30] Ingle L, Goode K, Carroll S, Sloan R, Boyes C, Cleland JGF (2007). Prognostic value of the VE/VCO2 slope calculated from different time intervals in patients with suspected heart failure. Int J Cardiol.

[31] Arena R, Myers J, Aslam SS, Varughese EB, Peberdy MA (2004). Peak VO2 and VE/VCO2 slope in patients with heart failure: a prognostic comparison. Am Heart J.

[32] Hartmann F, Packer M, Coats AJ, Fowler MB, Krum H, Mohacsi P (2004). NT-proBNP in severe chronic heart failure: rationale, design and preliminary results of the COPERNICUS NT-proBNP substudy. Eur J Heart Fail.

[33] Coeckelberghs E, Buys R, Goetschalckx K, Cornelissen VA, Vanhees L (2016). Prognostic value of the oxygen uptake efficiency slope and other exercise variables in patients with coronary artery disease. Eur J Prev Cardiol.

[34] Omland T, Sabatine MS, Jablonski KA, Rice MM, Hsia J, Wergeland R (2007). Prognostic value of B-type natriuretic peptides in patients with stable coronary artery disease: the PEACE trial. J Am Coll Cardiol.

[35] Van de Veire NR, Van Laethem C, Philippé J, De Winter O, De Backer G, Vanderheyden M (2006). VE/Vco2 slope and oxygen uptake efficiency slope in patients with coronary artery disease and intermediate peakVo2. Eur J Cardiovasc Prev Rehabil.

[36] De Schutter A, Lavie CJ, Patel DA, Artham SM, Milani RV (2013). Relation of body fat categories by Gallagher classification and by continuous variables to mortality in patients with coronary heart disease. Am J Cardiol.

[37] Lavie CJ, McAuley PA, Church TS, Milani RV, Blair SN (2014). Obesity and cardiovascular diseases: implications regarding fitness, fatness, and severity in the obesity paradox. J Am Coll Cardiol.

[38] Goel K, Thomas RJ, Squires RW, Coutinho T, Trejo-Gutierrez JF, Somers VK (2011). Combined effect of cardiorespiratory fitness and adiposity on mortality in patients with coronary artery disease. Am Heart J.

[39] Seki E, Watanabe Y, Shimada K, Sunayama S, Onishi T, Kawakami K (2008). Effects of a phase III cardiac rehabilitation program on physical status and lipid profiles in elderly patients with coronary artery disease: Juntendo Cardiac Rehabilitation Program (J-CARP). Circulation journal : official journal of the Japanese Circulation Society.

[40] Sato S, Makita S, Uchida R, Ishihara S, Majima M (2008). Physical activity and progression of carotid intima-media thickness in patients with coronary heart disease. J Cardiol.

[41] Byrkjeland R, Stensaeth KH, Anderssen S, Njerve IU, Arnesen H, Seljeflot I (2016). Effects of exercise training on carotid intima-media thickness in patients with type 2 diabetes and coronary artery disease. Influence of carotid plaques Cardiovasc Diabetol.

