# Correction to: Is Cardiorespiratory Fitness Related to Cardiometabolic Health and All-Cause Mortality Risk in Patients with Coronary Heart Disease? A CARE CR Study

**DOI:** 10.1186/s40798-019-0179-y

**Published:** 2019-02-05

**Authors:** Simon Nichols, Claire Taylor, Richard Page, Anna Kallvikbacka-Bennett, Fiona Nation, Toni Goodman, Andrew L. Clark, Sean Carroll, Lee Ingle

**Affiliations:** 1 Centre for Sport and Exercise Science, Sheffield Hallam University, Collegiate Hall, Collegiate Crescent, Sheffield, S10 2BP UK; 2 Carnegie School of Sport, Leeds Beckett University, Fairfax Hall, Headingley Campus, Leeds, LS6 3QS UK; 3 Sport Health and Exercise Science, Don Building, University of Hull, Cottingham Road, Hull, HU6 7RX UK; 4 Academic Cardiology, Castle Hill Hospital, Castle Road, Cottingham, HU16 5JQ UK; 5 City Health Care Partnership CIC, East Riding Community Hospital, Swinemoore Lane, Beverley, HU17 0FA UK

**Correction to: Sports Med Open**

**https://doi.org/10.1186/s40798-018-0138-z**

The original article [1] contains an error in the presentation of Table 4. Values denoting the significance of different variables were mistakenly omitted in the published version. The correct version of Table 4 can instead be viewed ahead.

**Table 4:** Cardiorespiratory fitness and physical activity characteristics expressed as mean (95% confidence intervals)

Variable	High CRF *n*=28	Mod CRF *n*=32	Low CRF *n*=10	Partial Eta Squared	*P*-value
VO_2Peak_ (ml/kg/min)	28.5 (27.3, 29.7)^+^	20.7 (19.5, 21.8)^+^	14.9 (12.8, 16.9)^+^	-	-
VO_2Peak_ (L/min)	2478.2 (2333.0, 2623.5)^+^	1749.0 (1613.1, 1884.9)^+^	1273.8 (1030.7, 1516.8)^+^	-	-
VO_2Peak_ - Lean (ml/kg/min)	45.2 (43.4, 47.0)^+^	34.8 (33.2, 36.5)^+^	26.8 (23.8, 29.8)^+^	0.670	<0.001^**^
VAT (ml/kg/min)	20.7 (19.3, 22.1)^+^	14.6 (13.3, 15.9)^+^	11.2 (8.9, 13.6)^+^	0.494	<0.001^**^
VE/VCO_2_ slope	30.1 (28.2, 32.1)^*✝^	37.4 (35.6, 39.2)^✝^	38.5 (35.2, 41.7)^*^	0.354	<0.001^**^
O_2_/HR (ml/beat)	17.0 (15.8, 18.2)^+^	13.8 (12.7, 14.9)^+^	11.3 (9.4, 13.3)^+^	0.311	<0.001^**^
OUES	2718.3 (2555.3, 2881.3)^*✝^	1963.5 (1811.1, 2116.0)^✝^	1699.0 (1426.2, 1971.7)^*^	0.485	<0.001^**^
eBR (%)	30.3 (23.6, 36.9)	28.1 (22.0, 34.3)	37.0 (26.0, 48.1)	0.028	0.384
Peak HR (bpm)	147 (141, 153)^*✝^	128 (122, 134)^✝^	119 (108, 129)^*^	0.308	<0.001^**^
Peak RER	1.13 (1.09, 1.12)^*^	1.09 (1.05, 1.12)^x^	0.97 (0.91, 1.04)^*x^	0.181	0.001^**^
Peak RPE	18 (17, 19)	18 (17, 19)	17 (15, 18)	0.072	0.083
1 Min HR Recovery (bpm)	-36 (-32, -40)^*^	-30 (-26, -34)^x^	-18 (-11, -25)^*x^	0.209	<0.001^**^
2 Min HR Recovery (bpm)	-54 (-50, -59)^+^	-45 (-40, -49)^+^	-32 (-25, -38)^+^	0.312	<0.001^**^
3 Min HR Recovery (bpm)	-60 (-56, -65)^+^	-49 (-45, -53)^+^	-37 (-30, -44)^+^	0.359	<0.001^**^
6 Min HR Recovery (bpm)	-67 (-62, -71)^+^	-54 (-50, -58)^+^	-41 (-33, -48)^+^	0.377	<0.001^**^
Exercise Test Duration (Secs)	963.2 (916.3, 1010.1)^+^	747.8 (703.9, 791.6)^+^	488.3 (409.8, 566.8)^+^	0.635	<0.001^**^
METs	8.1 (7.8, 8.5)^+^	5.9 (5.6, 6.2)^+^	4.3 (3.7, 4.8)^+^	-	-
Maximal CPET (%)	26 (93)	26 (81)	6 (60)		0.058
Achieves 150 Minutes of Moderate Activity Per-Week (%)	18 (64)^+^	9 (28)	5 (50)		0.011^**^
Achieves 75 Minutes of Vigorous Activity Per-Week (%)	7 (25)^+^	1 (3)	0 (0)		0.013^**^

*VO*_*2peak*_ Peak Oxygen Uptake, *VAT* Ventilatory Anaerobic Threshold, *VE/VCO*_*2*_ Ventilatory Efficiency with Respect to CO_2_ Elimination, *O*_*2*_*/HR* Oxygen Pulse, *OUES* Oxygen Uptake Efficiency Slope, *eBR* Estimated Breathing Reserve, *HR* Heart Rate, *bpm* Beats per Minute, *RER* Respiratory Exchange Ratio, *RPE* Rating of Perceived Exertion, *Secs* Seconds, *METs* Metabolic Equivalents

^**^Significant Group Effect

^*^Significant Difference Between High CRF and Low CRF

^✝^Significant Difference Between High CRF and Moderate CRF

^x^Significant Difference Between Mod CRF and Low CRF

^+^Significantly Different from all Other Groups
